# Insights into the Belgian Linguistic Conflict from a (Social) Psychological Perspective: Introduction to the Special Issue

**DOI:** 10.5334/pb.418

**Published:** 2017-11-21

**Authors:** Nicolas Van der Linden, Arne Roets

**Affiliations:** 1 Université Libre de Bruxelles, BE; 2 Ghent University, BE

**Keywords:** Belgium, intergroup relations, linguistic conflict, reconciliation

“If you think you understand the Belgian linguistic conflict, then obviously no one explained it properly to you” (Unknown).

In some way, Belgium is a contradiction. On the one hand, the country has greatly contributed to the political construction of Europe. On the other hand, it remains mired in the ongoing conflict between the two main linguistic groups: Flemings,^1^ who inhabit the northern part of the country and speak Dutch, and French-speakers or Francophones, who mainly live in Wallonia (the south of Belgium) and Brussels (which is geographically located in Flanders but is predominantly French speaking). This seemingly intractable conflict has culminated in two major recent political crises (in 2007–2008 and 2010–2011), which have fueled fears in some people that Belgium would eventually split (De Winter & Baudewyns, 2009; Rimé, Bouchat, Klein, & Licata, 2015).

Confronted with the dual movement of supranational integration and subnational regionalism, Belgian political institutions have been profoundly transformed since World War II. In response to requests stemming from both sides of the conflict, the Kingdom of Belgium has moved from a unitary to a federal state (Covell, 1986; Swenden & Jans, 2006). With 11 million inhabitants, it is now composed of three regions, which are defined on a territorial basis and deal with economic policy: Flanders, Wallonia, and Brussels representing, respectively, 58, 32, and 10% of the population. It also comprises three communities, which are defined on a linguistic basis and deal with cultural and educational policies: the Dutch-speaking community, the French-speaking community, and a small German-speaking community representing, respectively, approximately 56, 43.5, and .5% of the population. The Dutch- and French-speaking communities are both active in Brussels in a ratio of 1:9 (Hooghe, 2004).^2^ In other words, two layers of federal entities coexist within the same national territory. This reality is sometimes puzzling to the outside observer. Under the heading “Belgian is a small country with a complicated political structure”, *The Washington Post* (Titeca, 2017) even proposed to draw lessons from African politics in order to shed light on the apparent disorder prevailing in the land of surrealism.

Because of its intricate political and linguistic makeup, Belgium provides researchers interested in intergroup relations and intergroup conflict with a particularly rich field of investigation. However, Olivier Klein and Bernard Rimé, who convened a symposium titled “What has psychology to say about the Belgian linguistic conflict?” during the 2011 Annual Meeting of the Belgian Association for Psychological Sciences, noted that psychology in general, and social psychology in particular, still had to make a major contribution to the understanding of the Belgian linguistic conflict. This Special Issue aims to make a start in filling this void.

## Belgium: Historical and political contexts

The current dynamics of the Belgian linguistic conflict cannot be fully grasped without knowledge of the historical processes that created Belgium’s institutions and political culture. The history of the conflict can be divided into four episodes (Mnookin & Verbeke, 2009): Domination by the French-speaking elites, breakthrough of the Flemish movement, role-reversal in economic prosperity, and a changing institutional structure.

Belgium gained its independence from the Netherlands in 1830. Back then, the linguistic distribution of the population was very similar to the current one: a majority of Dutch-speakers, a minority of French-speakers, and a much smaller minority of German-speakers (Hooghe, 2004). However, the state was unitary and unilingual. Throughout the territory, the language of power and administration was French. Language use was also a marker of social status: In general, people speaking Dutch or German were from modest backgrounds, whereas people speaking French were from the upper classes or the nobility (Dassargues, Perrez, & Reuchamps, 2014).

In reaction to the domination of the French-speaking elites, which persisted until the 1960s, a Flemish movement was established (Vos, 2002). After years of struggle, it obtained the recognition of Dutch as an official language in 1898. Whereas its initial claim was a better recognition of Flemish culture and language within a unified Belgium, the Flemish movement evolved towards a sub-nationalism defined in ethnic terms (Martiniello, 1998).

The rise of Walloon nationalism came only after World War II (Hooghe, 2004). Until then, the steel and mining industries of Wallonia had been the engines of Belgium’s prosperity. However, after World War II, Wallonia’s economy started to decline, whereas the Flemish economy experienced a boom. The balance between the two regions’ gross domestic product per capita occurred in 1965. By the end of the 1980s, Flanders’ economy had established a considerable lead, and this gap was further widened in the 1990s. In reaction to this new situation, Walloon elites requested more autonomy in order to develop policies more adapted to the region’s struggling economy (Dassargues et al., 2014).

Requests for more linguistic and cultural autonomy from Flemish political parties and requests for more economic autonomy from French-speaking political parties provided the backdrop for the radical institutional transformations that have occurred in Belgium since the 1960s. In 1962 and 1963, legislation was approved which created a permanent language border and resulted in the division of the territory in three monolingual (i.e., Dutch, French, and German) areas (Vos, 2002). Within this legislation, Brussels received a special status as a bilingual area. The linguistic border was subsequently used to define the limits of the constituent parts (i.e., the communities and regions) of the Belgian federation. With time, more and more powers were transferred from the federal level to the communities and regions. Currently, French-speaking political parties consider these evolutions sufficient, whereas some Dutch-speaking political parties want more constitutional reforms. The latter request the abrogation of language facilities, which were granted to municipalities adjacent to the language border as part of the language legislation. Six of those currently crystallize the linguistic tensions (Hooghe, 2004). In these Flemish municipalities surrounding Brussels, French-speakers, who often form a considerable part of the population, have the right to deal with the authorities in their mother tongue. However, a more contentious issue is related to the control and allocation of governmental resources (Mnookin & Verbeke, 2009). Specifically, some Flemish representatives take issue with the fact that Wallonia receives more in terms of public expenditures than it contributes to the state revenues. In other words, they perceive that the “lazy” Walloons are unjustly benefiting from the hard earned wealth of their northern neighbors and call for the regionalization of national entitlement programs. The largest political party in both Flanders and Belgium, the Flemish nationalist N-VA, is at the forefront of these requests. Its president, Bart De Wever, has come to embody French-speakers’ fears that Belgium would eventually split (for an overview of the Belgian political landscape, see Meeusen, Boonen & Dassonneville, this issue).

The presentation of two other, overlapping, episodes allows drawing a more complete picture of the language divide in Belgium. Diverging representations associated with the two great wars have weighted heavily on relations between the two main linguistic groups (Vos, 2002). Although reality is much more nuanced, French-speakers usually believe that their ancestors were more often resistance fighters and Flemings more often collaborators during both wars, whereas Flemings usually believe that the repression of collaborators was more severe in Flanders than in Wallonia.^3^ Despite requests from Flemish representatives, no amnesty was granted to convicted collaborators up to now, a situation that continues to spur public debates (De Meulemeester, 2014; see also De Guissmé, Lastrego, Mélotte, and Licata, this issue).

The great wars transformed Belgium in another way. After World War I, German-speaking territories, located to the east of the country, were ceded from Germany to Belgium in compensation for losses and damages caused by the war. They were regained by Germany during World War II only to be ceded back to Belgium after the war. Because of (mostly alleged) sympathies and collaboration with the Nazi occupier, German-speakers were discriminated against (Dewulf, 2009). For instance, after the war, French became the only language in administration and education (Markusse, 1999). Charged with treason by their fellow Belgians and in a social climate of suspicion, they responded by showcasing themselves as “good” Belgians. They also isolated themselves from the other federal entities (Wagener, 2013). This has translated in two contrasting attitudes, the most prevalent being disinterest and distrust of politics, the other being aspiration for more autonomy. The latter attitude has led to requests that powers bestowed on the regions (e.g., economy) be transferred to the German-speaking community. Because of its small size and limited contribution to institutional reforms, the German-speaking community has usually been neglected in analyses of the Belgian linguistic conflict (see Luminet et al., 2012; Swenden, 2002). The article by Asbrock and Van Hiel (this issue) marks a sharp break with this trend.

## Belgium as a case study for (social) psychology

A fruitful strategy for investigating the dynamics of intergroup conflict involves considering these dynamics in a specific national context and across multiple levels of analysis (Pettigrew, 1998; Tajfel, 1982). Such requirements flow naturally from the recognition that the relevance, meaning, and intensity of constructs, as well as their embeddedness in a complex of influential factors, may vary in different (national) contexts (Vollhardt, & Bilali, 2008).

There are good reasons to single out Belgium as a case study. First, although the linguistic conflict has been intense at times, it has been confined to electoral competition and non-violent street protest (Hooghe, 2004). This opens up the possibility to study the dynamics of intergroup conflict in a setting where structural ‘violence’ (i.e., covert violence that harms individuals slowly through societal arrangements, such as the uneven distribution of resources and power) rather than direct violence (i.e., overt violence that involves immediate attacks on someone’s well-being and can quickly cause harm; Galtung, 1981) characterizes intergroup relations. Such approach has not been systematically adopted for some psychological concepts relevant to the study of intergroup conflict (e.g., collective victimhood; but see Jasini, Delvaux, Mesquita, this issue).

Secondly, Belgium is a multilingual society where members of the different linguistic communities not only live to a large extent in territorially distinct territories but also partake in different public spheres (Sinardet, 2009). Many factors have contributed to this state of affairs, starting with limited linguistic knowledge within the population: whereas more than half of the Flemish population has a good to excellent command of French, people having a good to excellent command of Dutch represent only 31 and 16% of the population in Brussels and Wallonia, respectively. Knowledge of German is even less widespread as people having a good to excellent command of this language represent only 19, 12, and 2% of the population in Flanders, Brussels, and Wallonia, respectively (Van Parys & Wauters, 2006).^4^ The institutional changes initiated in the 1960’s have also led to separations between Dutch-, French-, and German-speaking political and media systems. The once unitary political system broke into three linguistic segments between which there is no electoral competition. Except for Brussels and the facility municipalities, all Belgian geographical areas are under strictly monolingual regime. Hence, the political system provides no incentive to respond to the demands from the two other communities (Swenden, 2002). The same can be said about the media system, with each community having its own public broadcasting organization, while the commercial radio and television stations are also monolingual. Moreover, media report only to a limited extent on news about the other communities, and political debates about nationally relevant topics are conducted almost exclusively with representatives of the ingroup (Sinardet, De Swert, & Dandoy, 2007). The most tangible consequence of these breakdowns is that Belgian citizens know little or nothing about the other communities (Billiet, Maddens, & Frognier, 2006). According to Sinardet (2009), in such a segregated context, the development of a national identity is hardly possible, whereas attempts at (mis)representing the different Belgian linguistic communities as homogeneous groups with opposed public opinions are made easier, which creates a breeding ground for ethno-nationalist discourse. This context also has direct implications in terms of the type of threat that Flemings or French-speakers elicit in the eyes of the other group (see Meuleman, Abts, & Meeusen, this issue).

A third reason Belgium is worthy of selection as a case study for testing, revising or developing models of intergroup conflict is that it provides an ideal setting to examine relative groups status in flux. Indeed, a few studies have suggested that the relative status of the two main linguistic groups is not self-evident. From an objective standpoint, whereas Flemings are a majority in Belgium, they are a minority in Brussels, the opposite being true for French-speakers (Klein & Azzi, 2001). From a subjective standpoint, Klein et al. (2012) pointed out that some of the central traits of the Flemish stereotypes about French-speakers are typically associated with high-status groups. These traits, commonly found in surveys, are “arrogant”, “contemptuous”, “haughty”, or “feeling superior” (e.g., Nuttin, 1976). However, this high status association does not reflect a material reality: on most objective indices (numerical size, power, etc.), French-speakers constitute a lower-status group. One can only interpret these traits as a function of the frame of reference provided by a representation of Flanders’ history in which French-speakers “dominated” the region. Based on these and other observations, Klein et al. (2012) proposed that perceived relative status varies depending on which dimension of the conflict (linguistic vs. economic) is most salient at a given time (evidence substantiating this model is presented in Klein, Bouchat, Azzi, and Luminet, this issue). Such observations and analysis run counter to widely used psychological theorizing like social identity theory (Tajfel, 1982) or the stereotype content model (Fiske, Cuddy, Glick, & Xu, 2002), according to which the relative status is determined mainly, if not exclusively, by numerical size, status (or prestige), and power.

Individually, none of the previous characteristics is unique to the Belgian conflict. Canada and Switzerland, for instance, are also hosts to nonviolent conflicts between their linguistic communities, which, like Dutch-, French-, and German-speakers in Belgium, are living mainly in territorially distinct regions (Bougie, Usborne, de la Sablonnière, & Taylor, 2011; Stojanovic, 2009). And in the case of Northern Ireland, double minority and double majority models have been developed to account for the fact that the perceived relative status of Catholics and Protestants is elusive: The members of each group can feel or act as either minority or majority members depending on the interpersonal or intergroup context (Stevenson, Condor, & Abell, 2007). However, the combination of these characteristics probably makes for a unique conflict setting in Belgium.

## Review of past research

Although still relatively rare, some attempts by psychologists have been made to understand the dynamics of the Belgian intergroup conflict. These included studies on stereotypes and intergroup attitudes (Klein et al., 2012; Leyens & Yzerbyt, 1992; Mesquita, Delvaux, Klein, Licata, Mercy, & Rimé, 2010; Nuttin, 1976), (sub-)national identification (Rimé et al., 2015) citizenship representations (Duriez, Reijerse, Luyckx, Vanbeselaere, & Meeus, 2013; Meeus, Duriez, Vanbeselaere, & Boen, 2010; Sanchez-Mazas, Van Humskerken, & Casini, 2003), attributions (Klein & Licata, 2001), justice perceptions (Klein & Azzi, 2001; Klein et al., 2012), the impact of the media (Euwema & Verbeke, 2009), as well as collective memory and intergroup emotions (Alarcón-Henríquez, Licata, Leys, Van der Linden, Klein, & Mercy, 2010; Heenen-Wolff, Verougstraete, & Bazan, 2012; Klein et al., 2012; Mesquita et al., 2010; Rimé et al., 2015). Although the most frequent approach has been to use the Belgian linguistic conflict to answer questions and test predictions derived from psychological theories (e.g., Leyens & Yzerbyt, 1992), a few studies have taken the conflict as a starting point for their analysis (e.g., Klein et al., 2012).

In line with the results of studies in sociology and political sciences (e.g., Billiet, Maddens, & Frognier, 2006), analyses conducted by Rimé et al. (2015) revealed that Flemings identified less with Belgium and more with their region than French-speakers did. Besides variations in levels of identification, past research also documented differences in citizenship representations (Duriez, Reijerse, Luyckx, Vanbeselaere, & Meeus, 2013; Sanchez-Mazas, Van Humskerken, & Casini, 2003): whereas Flemings tend to define citizenship in ethnic terms, French-speakers tend to define it in civic terms. Moreover, two studies by Meeus, Duriez, Vanbeselaere, and Boen (2010) sought an explanation for the positive association usually observed among Flemings between subnational identification and outgroup derogation, and found support for a mediation hypothesis whereby identification with Flanders was associated with a more ethnic representation of citizenship, and consequentially a greater inclination to display ethnic prejudice.

Most of the recent research has concentrated on collective memory and intergroup emotions. Results of a recent comparative survey (Mesquita et al., 2010) found that Flemings and French-speakers punctuated the conflict differently: Flemings (particularly Flemish nationalists) tended to view the conflict as more ancient (median year of the onset of the conflict = 1830) than French-speakers (median = 1930), for whom the linguistic issue became a reality only when the Flemish movement radicalized. Both groups therefore view themselves as victims: Flemings as past victims of arrogant French-speakers; and French-speakers as present victims of dominant and nationalist Flemings. However, there was widespread consensus among respondents, irrespective of their mother tongue, that Flemings had suffered more from French-speakers than the other way around. Alarcón-Henríquez et al. (2010) further showed that recognition of shared past suffering can improve intergroup attitudes and lead to reconciliation between Flemings and French-speakers provided that intergroup trust is present. More recently, Rimé et al. (2015) tested the possibility that reconstructions of the past are shaped by current social conditions and interests. More specifically, they expected collective memories of victimization by French-speakers to fade among younger generations of Flemings because these collective memories would fail to provide an adequate account for the much-improved social conditions in which they are presently living compared to their ancestors. Surveying three generations of Dutch- and French-speakers, they observed generational differences consistent with their expectation: Younger generations demonstrated less consensus concerning the date of onset of the conflict, expressed a reduced perception of victimization of the in-group and an increased perception of victimization of the outgroup. Although present in the two language groups, this generational evolution was more pronounced among Flemish participants and was accompanied by corresponding changes in social identifications, intergroup attitudes, and political aspirations. Finally, closing the loop, Rimé et al. presented results suggesting that the decline in collective memories is the mechanism responsible for the lower levels of nationalist orientation observed among younger Flemings. These findings offer a possible fruitful perspective for understanding the recent decision of the N-VA not to put community issues high on its agenda during the next elections (Rousseau, 2017), a decision which has puzzled many observers (B. Rimé, personal communication, September 14, 2017).

In sum, psychological studies on the Belgian linguistic conflict, although limited in number and scope, revealed some differences (in e.g., (sub-)national identification or collective memory) between Flemings and French-speakers. More importantly though, this line of research also highlighted that social psychological differences between the linguistic groups are not fixed, as they evolve partly because of changing economic and structural circumstances. Evidence from Belgium also stressed the importance of taking account of the multiplicity of identities, as the very definition of the subgroups composing the country is far from self-evident.

## Summary of the contributions to the Special Issue

Building on this previous research, the different contributions to this Special Issue tackle three broad issues: 1) the differences and similarities in perspective of the different linguistic groups on specific, common issues, 2) attitudes and prejudices towards the other linguistic group(s), and 3) pathways to reconciliation between the members of the different communities.

With regard to the first theme, Klein, Bouchat, Azzi, and Luminet provide an inquiry into the differences between Flemish and Francophone citizens in the justice principles they endorse in the context of the linguistic conflict. In their two-wave longitudinal study, the authors demonstrate that such differences occur on two dimensions: language territoriality and distribution of resources. In particular, whereas among Flemish citizens (and a specific subgroup in particular), the principles of linguistic territoriality and an equity-based distribution of resources are dominant, principles of free choice in linguistic idiom and distribution based on need are most dominant among Francophone citizens. Of particular interest, these divergences are inflated in times of political conflict between the two communities, yet deflate in times of pacification.

In the second contribution within this theme, De Guissmé, Lastrego, Mélotte, and Licata reflect on the collective memories of the Flemish and Francophone communities. From this perspective, they direct their investigation to linguistic group differences with regard to attitudes towards World War II collaboration and amnesty. In two studies they demonstrate that although attitudes towards collaboration and amnesty are generally negative across groups, respondents in Flanders are less adamant in their condemnation, especially when they identified strongly with their linguistic group.

The third contribution investigates the differences and similarities between Flemish and Francophone citizens in the structure of their attitudes towards outgroups, including linguistic outgroups. As such, the study of Meeusen, Boonen and Dassonville also bridges to the second major theme in this Special Issue: intergroup stereotyping and prejudice between the different linguistic groups in Belgium. Their extensive investigation demonstrates that in citizens from both linguistic communities, negative attitudes towards the other linguistic group are part of an overarching generalized prejudice construct, which also incorporates prejudice towards other outgroups such as immigrants, homosexuals, and Jews. However, these specific prejudices have differential relationships with voting tendencies: whereas anti-immigrant feelings guide party preferences in both regions, negative attitudes towards the other linguistic group is only informative of party preference in Flanders, but has no informative value for citizens propensity to vote in Wallonia.

The similarities and differences between prejudices that specifically target the other linguistic group versus prejudice that targets immigrant groups are further dissected by Meuleman, Abts and Meeusen within a Flemish voter sample. In line with the previous contribution, the authors found a strong communality between anti-immigrant and anti-Francophone sentiment, with economic and cultural threat perceptions as a common basis. However, whereas regional ingroup identification was hardly relevant for anti-immigrant attitudes, it showed to be most relevant in explaining anti-Francophone attitudes.

Strong regional ingroup identification also proved to be an important mobilizing factor in the study of Jasini, Delvaux and Mesquita, who provide an extensive examination of the role of collective victimhood in explaining emotional responses and behavioral tendencies towards the perpetrating outgroup. In their study, the authors demonstrate that collective victimhood is negatively associated with intergroup affiliative emotions and positively with intergroup distancing emotions in Flemish as well as Francophone respondents. These emotional responses in turn predict behavioral tendencies of intergroup contact versus intergroup exclusion and revenge in both groups. As such, collective victimhood and its emotional correlates prove to be valuable to further our understanding of the dynamics of intergroup conflict, not only in violent confrontations, but also in in non-violent contexts such as the Belgian linguistic conflict.

Specifically focusing on the role of regional ingroup identification, Asbrock and Van Hiel investigate the unique and rarely studied German-speaking minority population of Belgium. Their research shows that minority members’ identification with their German-speaking community is associated with positive attitudes towards the community, without resulting in negative attitudes towards the two other communities. Disidentification with Belgium as a superordinate group, however, is associated with negative perceptions of all Belgian communities, the perception of severe inter-group conflict, and demands for dissolution of the Belgian federal state into independent regions. These results are particularly interesting because they show that a strong regional identity is not necessarily associated with negative attitudes towards other groups, but that especially disidentification with the superordinate (i.e., national) community may be problematic in this respect.

Finally, as a closing contribution to this Special Issue, Van Assche, Bostyn, De keersmaecker, Dardenne and Hansenne provide an investigation into the road to reconciliation between members of the linguistic communities, focusing on the role of cognitive style, ideology and intergroup emotions. In their work, the authors show that, in both Flemish and Francophone citizens, need for cognitive closure drives right-wing attitudes and essentialist thinking, which in turn is associated with less outgroup empathy and trust, and more outgroup anger. However, the presence of the positive emotions of outgroup trust and empathy, rather than the mere absence of outgroup anger proved to be critical as the affective basis for reconciliation.

## Concluding remarks and acknowledgments

The source of inspiration of the special issue was the above mentioned symposium during which the conveners, Olivier Klein and Bernard Rimé, called on Belgian psychologists to contribute their expertise in areas like “social identity”, “collective memories”, “prejudice, stereotypes, and discrimination”, and “emotions and communication” to the study of the intergroup issues raised by the Belgian linguistic conflict. To a certain extent, this call seems to have been heard (see Luminet et al., 2012). We hope this special issue will stand as another step in that direction.

Two experts in the respective field reviewed each article. When possible, we requested and received the contributions of one Belgian and one non-Belgian reviewer. We followed this procedure with the aim of striking a balance between contextual relevance and theoretical integration, between particularism and universalism. These reviewers were (in alphabetical order): Alejandra Alarcón, Boris Bizumic, Asteria Brylka, Ellen Delvaux, Stéphanie Demoulin, Kristof Dhont, Olivier Klein, Giovanna Leone, Christophe Leys, Olivier Luminet, Christina Matschke, Cecil Meeusen, Charles B. Stone, and Vincent Yzerbyt. We thank these reviewers for their invaluable contributions to this Special Issue.

## Footnotes

The term ‘Flemings’ is used interchangeably with the term ‘Dutch speakers’, although a minority of Flemings define themselves as French-speaking, whereas French speakers include Walloons as well as inhabitants of Brussels and its surroundings who generally do not consider themselves as Walloons (Deprez et al., 1996–97).

When reading these figures, one should bear in mind that it is very difficult to know the exact number of Dutch-, French-, and German-speakers because linguistic census is forbidden by law.

In this respect, recent research learned that collaboration within linguistic groups differed in kind rather than in degree. Whereas in Flanders, leaders of the Flemish movement collaborated with the promise they would be granted independence, in Wallonia, collaborators more often sought their self-interest. Evidence also suggests that repression was proportionally not more frequent in Flanders than in Wallonia (e.g., Beyen, 2002).

To the best of our knowledge, figures relating to linguistic knowledge of the members of the German-speaking community are not available.
